# Structure and origin of Tuvan gene pool according to autosome SNP and Y-chromosome haplogroups

**DOI:** 10.18699/VJGB-23-06

**Published:** 2023-03

**Authors:** V.A. Stepanov, N.A. Kolesnikov, L.V. Valikhova, A.A. Zarubin, I.Yu. Khitrinskaya, V.N. Kharkov

**Affiliations:** Research Institute of Medical Genetics, Tomsk National Research Medical Center of the Russian Academy of Sciences, Tomsk, Russia; Research Institute of Medical Genetics, Tomsk National Research Medical Center of the Russian Academy of Sciences, Tomsk, Russia; Research Institute of Medical Genetics, Tomsk National Research Medical Center of the Russian Academy of Sciences, Tomsk, Russia; Research Institute of Medical Genetics, Tomsk National Research Medical Center of the Russian Academy of Sciences, Tomsk, Russia; Research Institute of Medical Genetics, Tomsk National Research Medical Center of the Russian Academy of Sciences, Tomsk, Russia; Research Institute of Medical Genetics, Tomsk National Research Medical Center of the Russian Academy of Sciences, Tomsk, Russia

**Keywords:** gene pool, human population, genetic diversity, genetic components, Y-chromosome, Tuvans, генофонд, популяции человека, генетическое разнообразие, генетические компоненты, Y-хромосома, тувинцы

## Abstract

Tuvans are one of the most compactly living peoples of Southern Siberia, settled mainly in the territory of Tuva. The gene pool of the Tuvans is quite isolated, due to endogamy and a very low frequency of interethnic marriages. The structure of the gene pool of the Tuvans and other Siberian populations was studied using a genome-wide panel of autosomal single nucleotide polymorphic markers and Y-chromosome markers. The results of the analysis of the frequencies of autosomal SNPs by various methods, the similarities in the composition of the Y-chromosome haplogroups and YSTR haplotypes show that the gene pool of the Tuvans is very heterogeneous in terms of the composition of genetic components. It includes the ancient autochthonous Yeniseian component, which dominates among the Chulym Turks and Kets, the East Siberian component, which prevails among the Yakuts and Evenks, and the Far Eastern component, the frequency of which is maximum among the Nivkhs and Udeges. Analysis of the composition of IBD-blocks on autosomes shows the maximum genetic relationship of the Tuvans with the Southern Altaians, Khakas and Shors, who were formed during the settlement of the Turkic groups of populations on the territory of the Altai-Sayan region. A very diverse composition of the Tuvan gene pool is shown for various sublines of Y-chromosomal haplogroups, most of which show strong ethnic specificity. Phylogenetic analysis of individual Y-chromosome haplogroups demonstrates the maximum proximity of the gene pool of the Tuvans with the Altaians, Khakas and Shors. Differences in frequencies of Y-chromosome haplogroups between the Todzhans and Tuvans and a change in the frequencies of haplogroups from south to north associated with the East Asian component were found. The majority of the most frequent Y-chromosome haplogroups in the Tuvans demonstrate the founder effect, the formation age of which is fully consistent with the data on their ethnogenesis.

## Abstract (ru)

Тувинцы – один из наиболее компактно проживающих народов Южной Сибири, расселенный в основном на территории Тывы. Генофонд тувинцев является достаточно обособленным за счет эндогамии и очень низкой частоты межнациональных браков. Исследована структура генофонда тувинцев и других сибирских популяций по полногеномной панели аутосомных однонуклеотидных полиморфных маркеров и маркерам Y-хромосомы. Результаты анализа частот аутосомных SNP различными методами, сходства по составу гаплогрупп Y-хромосомы и YSTR-гаплотипов показывают, что генофонд тувинцев очень гетерогенен по составу генетических компонентов. Он включает в себя древний автохтонный енисейский компонент, доминирующий у чулымских тюрков и кетов, восточносибирский, преобладающий у якутов и эвенков, и дальневосточный, частота которого максимальна у нивхов и удэгейцев. Анализ состава IBD-блоков на аутосомах демонстрирует максимальное генетическое родство тувинцев с южными алтайцами, хакасами и шорцами, которые формировались при расселении тюркских групп популяций на территории Алтае-Саянского региона. Выявлен очень разнообразный состав генофонда тувинцев по различным сублиниям Y-хромосомных гаплогрупп, большинство из которых показывают сильную этническую специфичность. Филогенетический анализ отдельных Y-хромосомных гаплогрупп демонстрирует максимальную близость генофонда тувинцев с алтайцами, хакасами и шорцами. Внутри тувинского этноса обнаружены значительные различия между выборками из западных, южных и восточных районов Тывы по доле монгольского и енисейского генетических компонентов. Генетическое разнообразие тувинцев по Y-хромосомным гаплогруппам и максимально раз- нородный состав генетических компонентов свидетельствуют о самом высоком разнообразии тувинского генофонда по сравнению со всеми коренными народами Сибири. Обнаружены различия по частотам гапло- групп Y-хромосомы между тоджинцами и тувинцами и изменение частот гаплогрупп с юга на север, связан- ных с восточноазиатским компонентом. Большинство наиболее частых гаплогрупп Y-хромосомы у тувинцев демонстрирует эффект основателя, возраст формирования которых полностью согласуется с данными об их этногенезе

## Introduction

From the point of view of studying population and evolutionary genetic processes, analyzing genetic diversity, and reconstructing the genetic history of populations, the gene pool of the indigenous population of Southern Siberia is a unique system. The problems related to the analysis of the composition and ratio of various substrate components among the Siberian peoples, despite the high level of study, have a number of unanswered questions. In this regard, genetics provides the richest opportunities for studying these problems, since the development of new approaches to the analysis of the population gene pool makes it possible to bring ethnogenetic studies to a completely new level. Modern methods used in molecular genetic research and new bioinformatic developments make it possible to reliably identify various ancestral genetic components in the gene pool of various peoples and individuals.

One of the most important problems of ethnology and anthropology of the population of Southern Siberia is the issue of the formation of indigenous ethnic groups, in the solution of which, at present, methods of analyzing genomic data play an important role. The gene pool of the indigenous population of this region was formed due to the long-term and multi-stage mixing of a large number of local gene pools of various tribes of Caucasoid and Mongoloid origin. The indigenous ethnic groups of Southern Siberia are characterized by various anthropological types, complex ethnic and demographic history. The mixture of numerous Turkic, Mongolian, Yeniseian, Samoyed and Ugric groups based on the genetic substrate of the ancient Indo-European tribes and taiga Mongoloids formed as a result a motley picture of the genetic diversity of the population of this region (Gene Pool of the Population of Siberia, 2003).

The processes of merging and assimilation with the participation of various migration flows played an important role in the formation of modern Turkic-speaking populations of Southern Siberia, especially the Tuvans. In the era of the Eneolithic, Bronze and Early Iron Ages, the territory of Tuva was part of the habitat of the ancient Caucasoid population, which later developed the cultures of the Scythian-Siberian world (Alekseev, 1984). The penetration of the Central Asian Mongoloid component into the territory of Southern Siberia dates back to the VII–VI centuries BC. The appearance of the forest, taiga Mongoloid component also dates back to approximately the same time (Kiselev, 1951). Gradually, there was an increase in the Mongoloid component, from the predominance of the Caucasoid in the Scythian time to the formation in the XIII–XIV centuries AD of the modern Central Asian anthropological type of the Tuvans (Debets, 1948).

Tuvans are one of the most compactly living peoples of Russia, settled mainly in the territory of Tuva. In Russia, according to the All-Russian Population Census of 2010, the number of the Tuvans is 263,934 people. At the same time, the gene pool of the Tuvans is relatively isolated, due to endogamy and a very low frequency of interethnic marriages (Puzyrev et al., 1999; Kucher et al., 2003). The heterogeneity of the tribal composition of the Tuvans was shown (Potapov, 1969). For some groups of Tuvans, isolation of local populations was noted, caused both by geographical factors and historically, which is especially pronounced for Tuvans-Todzhans from the northeastern mountainous part of Tuva. In recent years, a number of scientific publications have been devoted to the study of the Tuvinian gene pool, which were focused on the study of the general spectrum of mtDNA lines, Y-chromosome haplogroups, and the detailing of individual clades (Stepanov, Puzyrev, 2000; Stepanov et al., 2001, 2006; Derenko et al., 2006; Kharkov et al., 2013; Damba et al., 2018а, b; Agdzhoyan et al., 2021).

The purpose of this study is a comprehensive analysis of the structure of the gene pool of Tuvans and the reconstruction of their origin in comparison with other populations of the indigenous population of Siberia. To address the issues of genetic proximity of Tuvans with other indigenous peoples, genotyping of a wide genomic set of autosomal markers using high-density biochips, as well as an expanded set of SNP and STR-markers of the Y-chromosome was performed in various indigenous peoples of Siberia.

## Materials and methods

The material of the study was DNA samples of men with a total number of 419 samples, representing the indigenous population of the Republic of Tuva. Samples were collected in the village Teeli (west of Tuva) (N = 44), village Kungurtug (south-east of the Republic) (N = 48), village Toora-Khem (north-eastern part of Tuva) (N = 23), and the city of Kyzyl (N = 304). Samples from Kyzyl were assigned to the corresponding territorial group according to the birthplaces of the donors. The samples were divided into five territorially distant groups: west (Barun-Khemchigsky, Bai-Taiginsky, Dzun- Khemchigsky, Sut-Kholsky, Mongun-Taiginsky districts) (N = 169), center (Chaa-Kholsky, Tandynsky, Kaa-Khemsky, Kyzyl, Ulug-Khem, Chedi-Khol, Piy-Khem, Tes-Khem, Ovyur, Erzin districts) (N = 179), east (N = 71), including the northeast (Todzhinsky district) (N = 23) and southeast (Tere-Kholsky district) (N = 48).

The sampling of primary biological material (venous blood) from donors was carried out in compliance with the procedure of written informed consent for the study. For each donor, a questionnaire was compiled with a brief pedigree, indicating ethnicity and places of birth of ancestors. An individual was assigned to a given ethnic group based on his own ethnic identity, his parents and place of birth.

For the analysis of Y-chromosome haplogroups and haplotypes of Tuvans, all 419 male DNA samples were used. For genotyping on chips, unrelated accessions from the village of Teeli of Bai-Taiga kozhuun (N = 28) were selected. Other populations of the indigenous population of Siberia are represented by: Chulyms, Khakas-Sagays, Khakas-Kachins, Southern Altaians, Kets, Khanty, Tomsk Tatars, Buryats, Yakuts, Evenks, Nivkhs, Udeges, as well as Kalmyks, Dungans and Kirghiz.

Genome-wide genotyping data were obtained using Infinium Multi-Ethnic Global-8 (Illumina) microarrays for SNP genotyping, including over 1.7 million markers. The material was deposited in the bioresource collection “Biobank of the Population of Northern Eurasia”. For comparative analysis, we used genotype data for 1677114 autosomal SNPs (Illumina Multi-Ethnic Global-8 biochip) of 917 samples and genotyping data for more than 3000 Y-chromosomal SNPs and 36 YSTRs from more than 1600 male samples representing the indigenous population of Siberia and neighboring regions. More than 30 population samples have been characterized, which are described in detail in our previous works (Kolesnikov et al., 2021, 2022). The NGSadmix method (Scotte, 2013) and the ADMIXTURE program (Alexander et al., 2009, 2011) were used to analyze the component composition and amount of impurities in individuals and populations, and a comparative analysis of autosomal SNP data and haplogroups and haplotypes of Y-chromosomes.

Autosomal SNP genotype array clustering and quality control were performed using a protocol developed by (Guo et al., 2014) using GenomeStudio (Ilumina. GenomeStudio, genotyping module v2.0.3), a software package that Illumina has developed for various genomic analyses. For filtering, normalizing and calculating standard genomic statistics and indicators, the standard set of programs, including vcftools, bcftools, and plink, proved to be optimal. To analyze linkage blocks identical in origin, the Refined IBD algorithm (Browning B.L., Browning S.R., 2013) was used, which shows more accurate results compared to the algorithms built into plink. The genotypes were preliminarily phased using the Beagle 5.1 software (Browning S.R., Browning B.L., 2007). To compare the populations, the sums of the average lengths of blocks identical in origin (IBD segments – identical by descent) were obtained between pairs of individuals.

To study the composition and structure of Y-chromosome haplogroups, two systems of genetic markers were included in the study: diallelic loci represented by SNPs and polyallelic highly variable microsatellites (YSTRs). With the help of 156 SNP markers, the belonging of the samples to different haplogroups was determined. The classification of haplogroups is given in accordance with the data of the International Society for Genetic Genealogy (website www. isogg.org). Analysis of STR haplotypes within haplogroups was performed using 44 STR markers of the non-recombining part of the Y-chromosome (DYS19, 385a, 385b, 388, 389I, 389II, 390, 391, 392, 393, 426, 434, 435, 436, 437, 438, 439, 442, 444, 445, 448, 449, 456, 458, 460, 461, 481, 504, 505, 518, 525, 531, 533, 537, 552, 570, 576, 635, 643, YCAIIa, YCAIIb, GATA H4.1, Y-GATA-A10, GGAAT1B07).

STR markers were genotyped using capillary electrophoresis on an ABI Prism 3730 genetic analyzer. Genotyping of SNP markers was performed using PCR and subsequent analysis of DNA fragments using RFLP analysis. Experimental studies were carried out on the basis of the Center for Collective Use of Research Equipment “Medical Genomics” (Research Institute of Medical Genetics of the Tomsk National Research Medical Center). The construction of median networks of Y-chromosome haplotypes was carried out using Network v.10.2.0.0 (Fluxus Technology Ltd; www. fluxus-engineering.com) using the Bandelt median network method (Bandelt, 1999). The generation age of the observed diversity of haplotypes in haplogroups was estimated using the ASD method (Zhivotovsky, 2004), based on the mean square differences in the number of repeats between all markers.

## Results and discussion

Genotyping of a large array of SNPs makes it possible to study in great detail the patterns of haplotype diversity that mark various substrate and superstrate layers of the population gene pool, the degree of miscegenation with the alien population at various levels – from individual to generic and ethnic, to conduct a detailed analysis of the demographic history of various populations and analyze the molecular phylogenetic and phylogeographic structure of Y-chromosome haplogroups. This makes it possible to more accurately reconstruct the genetic and demographic events that occurred in the past. The use of modern bioinformatic approaches on a wide array of SNPs and a detailed phylogeny of uniparental lines makes it possible to more accurately reconstruct the formation of the Tuvan gene pool.

After processing the data on the results of a microchip study to filter the progenotyped samples and carry out further calculations, a search was first made among the Tuvans of mestizos using the NGSadmix program. The NGSadmix method, when launched on the data array that we formed, showed that all progenotyped samples of the Tuvans do not have crossbreeding, which is fully consistent with the data of the DNA donor questionnaire. The obtained data on the frequencies of SNPs in the studied population samples were used to elucidate the genetic relationships between different ethnic groups. The ADMIXTURE algorithm was used to reduce the dimension and identify the genetic components.

Component composition of the gene pool of the Tuvans

To identify individual genetic components in the gene pool of the studied populations, the ADMIXTURE program was used, which makes it possible to identify the mixed composition of a set of individuals based on genotype data and, thereby, to make assumptions about the origin of the population. Modeling using ADMIXTURE has recently become one of the main methods of analysis in the study of the gene pools of modern and ancient human populations, allowing you to analyze the same data at different hierarchical levels.

Tuvans, in comparison with most Siberian populations, show a very diverse composition of genetic components Their distribution is most clearly manifested at K = 12. For almost all Siberian populations, the complete dominance of one genetic component, characteristic of individual samples or closely related indigenous peoples, is shown. In addition to the Tuvans, a rather heterogeneous component composition was also found among the Khakas-Kachians. The spectrum of genetic components of the Kachins almost completely coincides with the Tuvans, but differs in their proportions

Altai component. With the maximum frequency in the Tuvans (53 %), the genetic component that dominates in the Southern Altaians (up to 90 %) is represented. Taking into account the fact that the analyzed sample of the Tuvans represents the westernmost region bordering the Republic of Gorny Altai, this is quite natural. It is presented with sufficient frequency among the Kyrgyz (9.8 %) and Khakas-Kachins (7.6 %), related to the Southern Altaians. Probably, this genetic component is associated with the influence of Turkic speakers in the formation of modern South Siberian peoples. Previously, the proximity of the Altaians and Tuvans was shown by analyzing the allele frequencies of the ZFX gene (Khitrinskaya et al., 2010), X-linked STR markers (Vagaitseva et al., 2014), enzymes and blood proteins (Spitsyn et al., 1984), frequencies blood groups of the ABO system and according to their anthropological parameters (Bogdanova, 1978a, b; Alekseev, 1984; Alekseeva, 1984).

East Siberian component. The second most common among the Tuvans is the East Siberian genetic component (21 %), which is dominant among the Yakuts (94 %), Evenks from Yakutia (93 %) and Transbaikalia (62 %). This corresponds to the linguistic data on the South Siberian origin of the ancestors of modern Yakuts. It is 30 % among the Buryats, 12 % among the Kachins, and 4 % among the Southern Altaians. The distribution of this genetic component is consistent with the classification of racial types. Tuvans, Tofalars, Yakuts and Dolgans are carriers of the traits of the North Asian minor race – one of the subdivisions of the continental branch of the great Mongoloid race. Two moderately different types are distinguished in the composition of the North Asian Mongoloids – Baikal and Central Asian. The first type is typical primarily for the Tungus-Manchurian peoples, the second – for the Turkic and Mongolian peoples (Turkic Peoples of East Siberia, 2008).

East Asian component. In third place in the Tuvans (11 %) is the dominant component of the Dungans (91 %), Buryats (63 %) and Kalmyks (54 %). It manifests itself most clearly at K = 12. It makes up a larger proportion among the Kirghiz (49 %), Kazakhs (46 %), Uzbeks (43 %), Khakas-Kachins (41 %) and Tomsk Tatars (24 %) and has a small share among the Kachins (4 %) and Southern Altaians (4 %). It is this genetic component that reflects the contribution of the latest groups of immigrants from the territory of Mongolia to the gene pool of the population of Southern Siberia. Almost all other studied populations of Siberia and the Far East – the Yakuts, Shors, Khakas-Sagays and Chulyms demonstrate the almost complete absence of this component. It was not found among the Evenks, Khanty, Kets, Chulyms, Chukchi, Koryaks and Nivkhs. The general picture of the distribution of this genetic component is in good agreement with anthropological and ethnographic data on the influence of the Mongol expansion on the ethnogenesis of the studied ethnic groups

Yeniseian component. The largest share of this component is characteristic of the Chulym Turks (94 %) and Kets (65 %). In the Kets, its proportion is lower due to miscegenation and the detection by the NGSAdmix method of a recent Caucasoid admixture in many samples. Among Tuvans, its frequency is 6.9 %, and among Kachins, 20 %. The results obtained are in good agreement with the data of ethnology, anthropology and linguistics on the contribution of the Yeniseian component to the formation of various peoples of the Altai-Sayan region and the historical areas of the Yeniseian languages

Far Eastern component. The last genetic component in the Tuvans present with a significant frequency (4.9 %) prevails in the Nivkhs (96 %) and Udege (56 %). It is present with a low frequency among the Trans-Baikal Evenks (11 %), Buryats (10 %), Kalmyks (8 %) and Dungans (6 %). Probably, its presence reflects the contribution of the taiga Mongoloids, who in ancient times settled westward from Primorye and Transbaikalia.

It can be assumed that the Samoyed component can also be present in the Tuvinian gene pool, however, its determination requires an analysis of the population groups in which it is dominant (Nenets, Enets, Nganasans and Selkups).

Identical in origin clutch blocks. As a result of bioinformatics processing of genotyping data from high-density biochips of various Siberian populations, an analysis was made of the coincidence of DNA fragments common in origin between populations and individuals. A segment with identical nucleotide sequences is IBD in two or more individuals if they inherit it from a common ancestor without recombination, that is, in these individuals the segment has a common origin. The expected length of an IBD segment depends on the number of generations since the last common ancestor. One of the applications of the analysis of genome regions of common origin is the quantitative assessment of the degree of relationship between individuals, which can also supplement information on the genetic relationships of populations (Gusev et al., 2011).

Samples from the sample of the Tuvans showed the maximum match in IBD blocks among themselves (10.07 %), then with a sample of the Southern Altaians (1.62 %), Evenks (0.81 %), Yakuts (0.77 %), Chulyms ( 0.70 %), Khakas-Sagays (0.66 %), Khakas-Kachins (0.64 %), Buryats (0.58 %), Kalmyks (0.57 %), Udeges (0.39 %) and Khanty (0.38 %). The degree of overlap of IBD blocks between the Tuvans and other population samples is consistent with the results of ADMIXTURE on the distribution of allele frequencies and common genetic components in these populations. The FROH inbreeding coefficient was also calculated for all individuals by homozygosity blocks (ROH). For the Tuvans, its value (0.0151) is much lower than for the Chulyms (0.0292), Kazyms (0.0280) and Russkinskaya Khanty (0.0266), Kets (0.0259) and Khakas-Sagays from the foothill Tashtyp region. Almost equal to the Tuvans in terms of FROH value are the samples of the Southern Altaians (0.0168) and the Khakas- Kachins of the Shirinsky district (0.0146). This indicates the absence of a significant role of inbreeding in the formation of the gene pool of modern Tuvan populations.

Haplogroups of the Y-chromosome

For the most frequent Asian haplogroups of the Y-chromosome in the Tuvans, additional terminal SNPs were genotyped, which made it possible to more accurately separate the samples into individual specific sublines. The frequencies of occurrence are indicated only for them (see the Table). The frequencies of other rather rare haplogroups represented by separate samples, indicated in an earlier article (Kharkov et al., 2013), are not given here, since additional SNPs were not selected for them

**Table 1.**
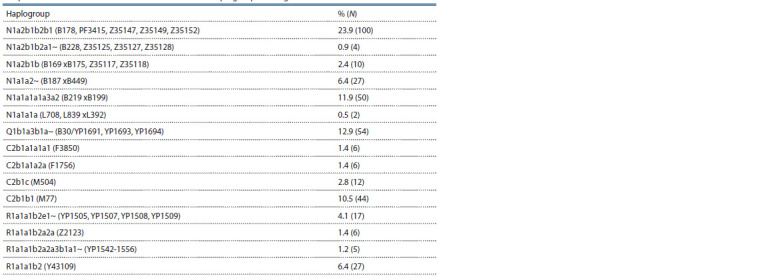
Frequencies of occurrence of the main Y-chromosome haplogroups among the Tuvans

**Fig. 1.**
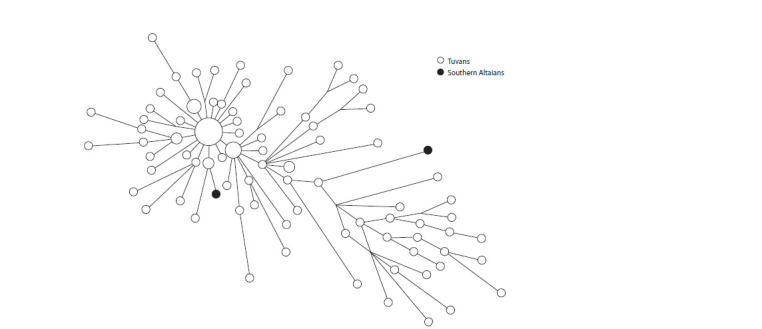
Median network of YSTR haplotypes of the N1a2b1b2b1 haplogroup in Tuvans and Southern Altaians.

The most frequent Y-chromosome haplogroup in the Tuvans is N1a2b1-B169, which makes up 24 % of the total array of male samples. It is divided into three sublines that differ in terminal SNPs and haplotype clusters. Its variant N1a2b1b2b1 (B178, PF3415, Z35147, Z35149, Z35152) is present with the maximum frequency among the Tuvans. In addition to the Tuvans, two samples of the Southern Altaians belong to it. According to the YFull website, this line was also found in one man from Kyrgyzstan and two from China. The haplotypes of this lineage have a stellar phylogeny, indicating a strong founder effect (Fig. 1).

The age of this line among the Tuvans according to YSTR is 1442 years (SD = 368 years). Its presence among the Altaians, Kirghiz and Chinese in the form of single samples is possibly associated with the inclusion of individual men of Tuvan origin in their composition. This line among the Tuvans represents a common genetic substrate for them, which is unequivocally connected with the heritage of the Samoyed population of the territory of Southern Siberia. The presence of different ethnospecific variants of the N1a2b1 haplogroup among the Tuvans, Khakas, and Shors indicates a significant genetic differentiation between them. This confirms the absence of migrations of carriers of this haplogroup and gene exchange over the past few hundred years. The main factor in its spread on the territory of Tuva was the genetic isolation of local Samoyedic groups and the intensive increase in their population. Four samples of Tuvans belong to a very rare parallel line N1a2b1b2a1~ (B228, Z35125, Z35127, Z35128). It was previously found in Mongols (Illimäe et al., 2016). The third Tuvan subline (хB175, Z35117, Z35118) includes 10 samples.

**Fig. 2.**
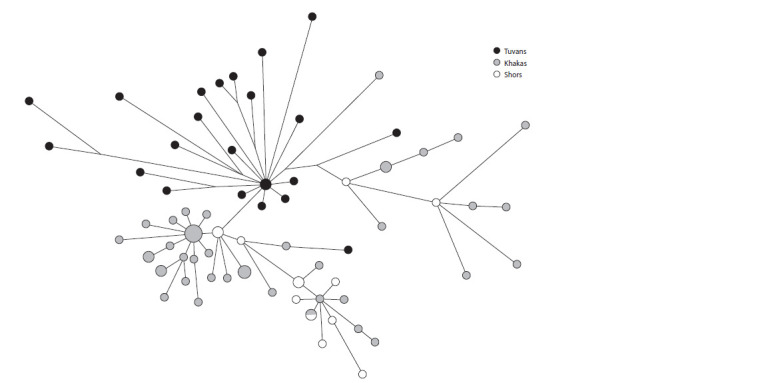
Median network of YSTR haplotypes of the N1a2b1b2b1 haplogroup in Tuvans and Southern Altaians.

The second most frequent among the Tuvans is the haplogroup N1a1 (19 %), which is divided into three branches. In the total sample, its frequency is inferior to N1a2b1 by only 5 %, covering slightly less than 30 % of samples in the west of Tuva. The first line N1a1a2~ (B187 xB449) in the total sample of the Tuvans has a frequency of 6.4 %. In the eastern regions – Todzhiinsky and Tere-Kholsky, this haplogroup was not found. This variant is very ethnospecific and is not found in other populations. The sister line parallel to it (N1a1a2 ~ B499) with a relatively recent divergence from the Tuvan line is also characteristic of the Khakas-Sagais and Shors. It dominates in frequency in the Khakas seoks Khyi and Khobyi. Among the Shors, this haplogroup includes all men of the seok Kyi and Kobyi (Kharkov, 2020). On the median networks, the haplotypes of these lines in the Tuvans, Khakas, and Shors form three clusters that do not intersect with the Tuvans, except for one sample (Fig. 2).

At the same time, the haplotypes of the Khakas-Sagays of the Tashtyp district, bordering Shoria, are very close to the Shors and demonstrate a strong recent founder effect. The total age of the Tuvan haplotype cluster was 1863 years (SD = 294 years). This shows the long-standing division of these lineages between the Tuvans, Khakas, and Shors, and rather strong founder effects for individual seoks of Khakas and Shors. This subline has a very limited geographic range. Most likely, the initial place of its distribution was the territory of Tuva, from where it spread to Gornaya Shoria and then to Khakassia. It separated from the main stem of N1a1 very early and, like many other rare Y-chromosomal lineages, was preserved with a sufficiently high frequency only in relatively isolated mountain populations. Its separation from the main stem of the N1a1a haplogroup occurred approximately 10,700 years ago (YFull). Due to its population specificity and isolation, the relationship of this variant with the Samoyedic, Ugric, or other genetic components is ambiguous.

The second subline N1a1a among the Tuvans is N1a1a1a1a3a2 (B219 xB199). It is represented in all districts and has a frequency of 11.9 %. It also includes three samples of the Altaians. It was not found among the Khakas, Shors and Chulyms. The line N1a1a1a1a3a2c2-B199, which is closely related to it, dominates in the Eastern Buryats and is represented by a rather high frequency in the Western Buryats. The appearance of these lines is unambiguously connected with the settlement of Mongolian ethnic groups in Tuva, Buryatia and Altai. The age of this line according to haplotypes among the Tuvans was 1500 years (SD = 304 years). The spread of this Y-chromosome lineage occurred a little later than the carriers of the Tuva-Shor-Khakas branch N1a1a2~.

Only two specimens of Tuvan-Todzhans belong to a very rare lineage N1a1a1a (L708, L839 xL392). In terms of haplotypes, it is very close to the Yakut-Evenki haplogroup N1a1a1a1a4a1a1, but is not mutated in its terminal SNPs (M1979, M1984, M1988, M1991). The presence of this Y-chromosome variant in the Todzhans is consistent with the distribution of the East Siberian genetic and overlap in IBD blocks with the Yakuts and Evenks. This lineage also includes four samples of Khakas-Sagay men from the Askizsky district with haplotypes close to those of Tuva.

**Fig. 3.**
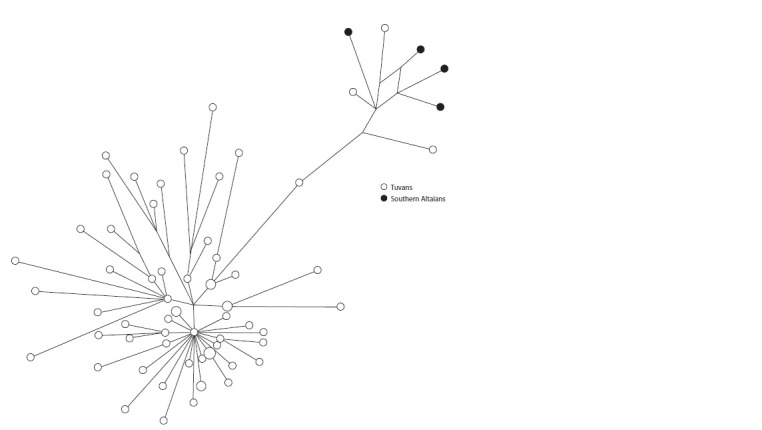
Median network of YSTR haplotypes of haplogroup Q1b1a3b1a~ – B30 in Tuvans and Southern Altaians.

Haplogroup Q1b1a3b1a~ (B30/YP1691, YP1693, YP1694) occupies 13 % of the total sample of the Tuvans. Its maximum frequency falls on the eastern samples of the Todzhans and Tuvans of the Tere-Kholsky kozhuun (25 %). Four samples of Southern Altaians also belong to this lineage (Fig. 3).

The descending gradient of this haplogroup from east to west was shown on the territory of Tuva earlier (Kharkov et al., 2013; Damba et al., 2018b; Agdzhoyan et al., 2021). The highest frequency of the haplogroup Q1b1a3b1a~ for Tuvans in Todzha is apparently a consequence of their relative genetic isolation and the preservation of a greater proportion of the local autochthonous Yeniseian genetic component. The age of this line according to haplotypes among the Tuvans was 2187 years (SD = 446 years). The distribution frequency of the haplogroup Q1b1a3b1a~ and its related lines Q1b1a3b1a2- B33 and Q1b1a3b4-B31 in the populations of the indigenous peoples of Southern and Western Siberia reflects the contribution to their gene pools of local aboriginal population groups belonging to the Yeniseian language family, which are quite ancient in origin. Analysis of the Y-chromosomal sublines Q1b1a3b shows that the original center of origin and settlement of its carriers is the territory of modern Tuva.

Different populations with a share of the Mongolian genetic component have different haplogroups and sublines, the origin of which is associated with the settlement of various ethnic groups and migration events of different times. Among the Tuvans, the result of the Mongolian contribution, in addition to N1a1a1a1a3a2, is the haplogroups of the clades C2b1, O2 and O3. All of them are very close to the variants presented with a high frequency among the Mongols, Buryats and Kalmyks. The share of C2b1c (M504) and C2b1a1a1a1 (F3850) is the highest in the southeastern sample (15 %). Lineage C2b1a1a1a1 (F3850) was found only in the southern and southeastern regions. The more frequent line C2b1b1 (M77) shows a clinal decrease in frequency from southeast to west. The same is true for haplogroups O2 and O3. In the gene pool of almost all the populations studied, in which the Mongolian genetic component is not detected by autosomal SNP, the indicated Y-chromosome haplogroups are also absent. Phylogenetic analysis of Y-chromosomal sublines and haplotypes shows that the center of origin and distribution of the carriers of the Mongolian component is the territory of Central Asia

These haplogroups among the Tuvans are a legacy of the genetic contribution of late Mongoloid migrants, reflecting the contribution of the Xiongnu and Mongolian settlers to the territory of Tuva. Thus, genetic data confirm that the penetration of Mongolian nomads into the territory of Tuva came from the south, gradually spreading to the northern regions, and, accordingly, the mongolization of the population of Tuva was most pronounced precisely in the southern regions. This coincides with the data of paleoanthropology (Alekseev, 1984) and anthropology of the modern population (Bogdanova, 1978a). The data of linguistics characterizing the southeastern dialect as formed as a result of the significant influence of the Mongolian language also completely coincide with the distribution of this component and the frequencies of haplogroups that we obtained.

The haplogroup R1a1a (12 %) among the Tuvans includes seven different lines. Six Tuvan men belong to the R1a1a1b2a2a (Z2123) lineage. Three Altaians and two Kirghiz also belong to it. Five more Tuvans belong to the line R1a1a1b2a2a3b1a1~ (YP1542, YP1556) close to it. It dominates in frequency among the southern Altaians and Teleuts. Twenty-seven Tuvans had the R1a1a1b2 (Y43109) line, which was divided into three variants differing in haplotypes. Sixteen Tuvans and five Southern Altaians belong to one variant. In the second, there are four Tuvans, Khakas from the Turan and Khyzyl Khaya seoks, and almost all Shors from the Tartkyn, Shor-Kyzai and Kara-Shor seoks. In the third, there are seven Tuvans, Khakas from various seoks of the Beltir and Biryusin, and Shors of the seoks of the Cheley and Chediber. This confirms the data that some groups of Tuvans who roamed in the Minusinsk Basin and were later called the “Beltyr” were completely assimilated by local tribes, constituting one of the components of the formation of the ethnos of modern Khakas.

The haplogroup R1a1a1b2e1~ (YP1509) among the Tuvans is also divided by haplotypes into two lines. Nine samples of Tuvans of the first variant are very similar in haplotypes to this variant among the Khakas of the Kharga seoks and the Shors of the Karga and Cheli seoks and one Teleut. Eight samples belong to another specific variant common among the Telengits and Northern Altaians.

A very large diversity of the R1a1a haplogroup was shown among the indigenous population of the Altai-Sayan region. Its various sublines split long ago and show no star-like haplotype phylogeny, reduced diversity, or traces of the founder effect. This indicates a significant size of the effective size of the populations of the ancient Caucasoids and Turks, who introduced these components into the gene pool of modern Tuvans, Khakas and Shors. Founder effects with significant demographic growth were found only in the Southern Altaians, Kirghiz and Teleuts in the haplogroup R1a1a1b2a2a3b1a1~. The distribution of various discovered sublines of the haplogroup R1a1a in the territory of Tuva, Altai, Khakassia and Shoria is most likely associated with the Turks and the Yeniseian Kyrgyz.

Of the other haplogroups among the Tuvans, eight more are single samples (D, E, I1, I2a, J1, J2a, J2a1 and R1b). Most likely, their presence is partly due to the recent miscegenation and earlier dispersal of the Central Asian populations. The results of the study of the detailed phylogeny of Y-chromosome haplogroups made it possible to more accurately analyze the component composition of the Tuvan gene pool. This is a more accurate addition to the analysis of autosomal markers, which makes it possible to reconstruct in detail the formation of their gene pool. This information is also important for describing the similarities and differences between the compared groups, as well as the processes of their ethnogenesis. Various Y-chromosome haplogroups in the Tuvan gene pool demonstrate their genetic affinity with the Altaians, Khakas, Shors, Buryats, Mongols, Evenks, Kets, Chulym Turks, and Teleuts. This allows us to characterize in more detail the gene pool of the indigenous South Siberian population and the genetic relationships and continuity of populations living in this territory.

## Conclusion

Thus, in the present study, a detailed study of the gene pool of Tuvans was carried out based on the data of high-density biochips and a wide range of SNPs of the non-recombining part of the Y-chromosome. A very heterogeneous composition of the gene pool of the Tuvans and Khakas was found, both in autosomal SNPs and in various sublines of Y-chromosomal haplogroups. The maximum closeness of the gene pool of the Tuvans with the Altaians, Khakas and Shors is shown. Analysis of IBD blocks and individual rare variants of male lines demonstrates traces of more ancient connections with the ancient aboriginal population of this region and the populations of Eastern Siberia and the Far East. Within the Tuvan ethnos, significant differences were found between samples from the western, southern, and eastern regions of Tuva in terms of the proportion of the Mongolian and Yeniseian genetic component. The genetic diversity of the Tuvans in Y-chromosomal haplogroups and the most heterogeneous composition of genetic components indicate the highest diversity of the Tuvan gene pool, compared to all other indigenous peoples of Siberia

In the future, we plan to analyze in more detail the structure of the gene pools of the South and West Siberian populations by adding population samples of the Samoyedic peoples – the Nenets and Selkups.

## Conflict of interest

The authors declare no conflict of interest.
